# Supplementary material for: Differentiating axonal loss and demyelination in chronic MS lesions: A novel approach using single streamline diffusivity analysis
Source: PLoS One. 2021 Jan 6;16(1):e0244766. doi: 10.1371/journal.pone.0244766 (PMC7787472; doi:10.1371/journal.pone.0244766)
Supplement: S1 Fig — a. Diffusivity values in 200 individual voxels randomly selected in brain’s white matter of normal subject. While AR and RD vary significantly (Coefficient of variability: AD-21%, RD-26%) and in opposite directions, MD remains relatively constant (Coefficient of variability: MD-7%). RD values multiplied by 2. b. Correlation between AD and RD in individual voxels randomly selected in brain white matter of normal subject (r = 0.85, p<0.001). (DOCX) [file pone.0244766.s001.docx]

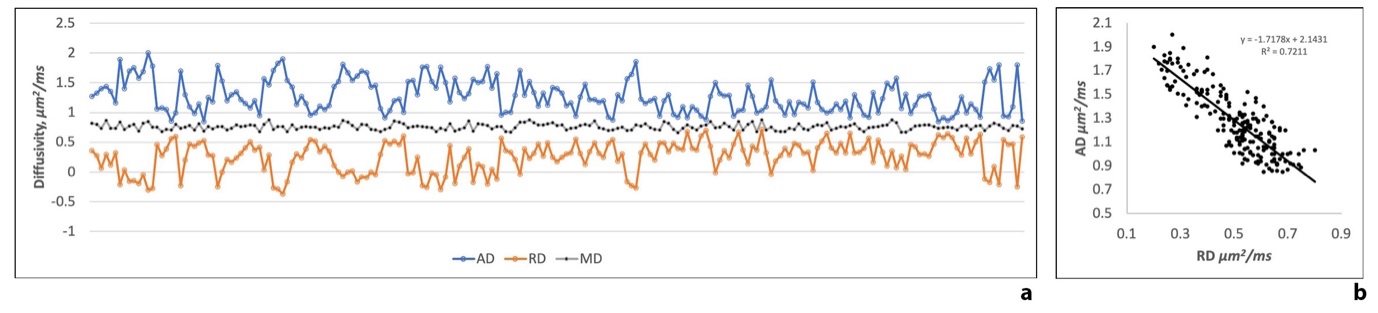


*S1 Fig. MD, AD and RD values in normal white matter.*

1. *Diffusivity values in 200 individual voxels randomly selected in brain’s white matter of normal subject. While AR and RD vary significantly (Coefficient of variability: AD-21%, RD-26%) and in opposite directions, MD remains relatively constant (Coefficient of variability: MD-7%).*

*RD values multiplied by 2.*

*b. Correlation between AD and RD in individual voxels randomly selected in brain white matter of normal subject (r=0.85, p<0.001).*
