# Supplementary material for: Differentiating axonal loss and demyelination in chronic MS lesions: A novel approach using single streamline diffusivity analysis
Source: PLoS One. 2021 Jan 6;16(1):e0244766. doi: 10.1371/journal.pone.0244766 (PMC7787472; doi:10.1371/journal.pone.0244766)
Supplement: S2 Fig — a. Left column demonstrates original AD and RD profiles of lesional (blue) and non-lesional (red) fibers. Blue arrow indicates linear fitting of lesional fiber. Red arrow indicates linear fitting of non-lesional fibers. Horizontal axis: points along the fibers. Point 7 indicates lesion. Vertical axis: μm2/ms. b. Slopes of ΔAD, ΔRD and ΔMD in individual lesional/non-lesional pair. (DOCX) [file pone.0244766.s002.docx]

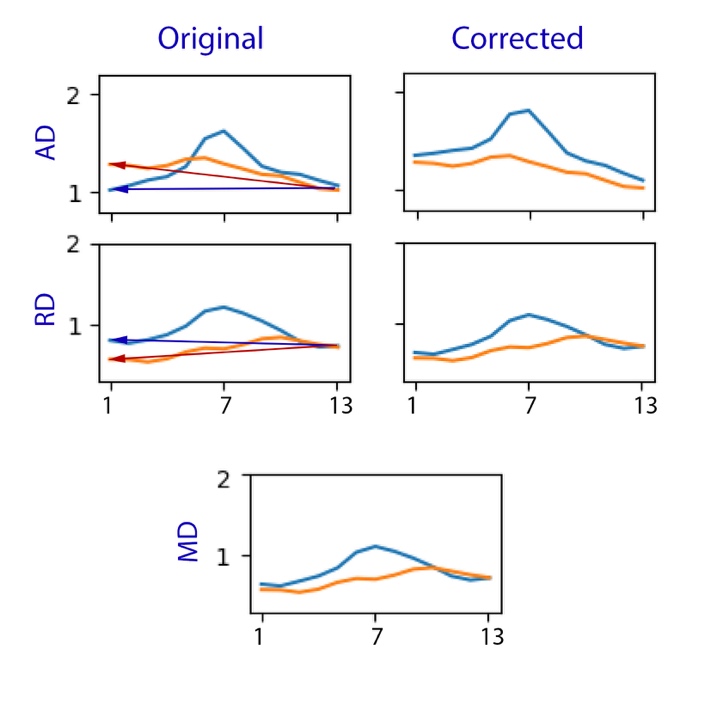


S2 Fig a


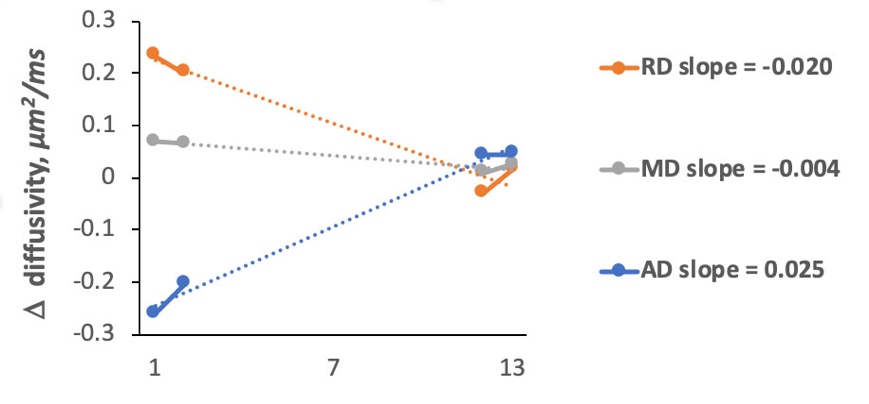


S2 Fig b.

*S2 Fig. MD-based normalisation.*

1. *Left column demonstrates original AD and RD profiles of lesional (blue) and non-lesional (red) fibers. Blue arrow indicates linear fitting of lesional fiber. Red arrow indicates linear fitting of non-lesional fibers. Horizontal axis: points along the fibers. Point 7 indicates lesion. Vertical axis: μm^2^/ms*
2. *Slopes of* Δ*AD,* Δ*RD and* Δ*MD in individual lesional/non-lesional pair.*
