# Supplementary material for: Differentiating axonal loss and demyelination in chronic MS lesions: A novel approach using single streamline diffusivity analysis
Source: PLoS One. 2021 Jan 6;16(1):e0244766. doi: 10.1371/journal.pone.0244766 (PMC7787472; doi:10.1371/journal.pone.0244766)
Supplement: S1 File — (DOCX) [file pone.0244766.s003.docx]

1. The following sequences were acquired using a 3T GE Discovery MR750 (GE Medical Systems, Milwaukee, WI scanner) in following order:
2. Pre-contrast Sagittal 3D T1: GE BRAVO sequence, duration 4 min each, FOV 256mm, Slice thickness 1mm, TE 2.7ms, TR 7.2ms, Flip angle 12°, Pixel spacing 1mm. Acquisition Matrix (Freq x Phase) is 256x256, which results in 1mm isotropic acquisition voxel size. The reconstruction matrix is 256x256.
3. FLAIR CUBE; GE CUBE T2 FLAIR sequence, duration 6 min, FOV 240mm, Slice thickness 1.2mm, Acquisition Matrix (Freq x Phase) 256x244, TE 163ms, TR 8000ms, Flip angle 90°, Pixel spacing 0.47 mm. The reconstruction matrix is 512x512.
4. Echo-Planar Imaging based diffusion weighted MRI, duration 9 min (64-directions with2mm isotropic acquisition matrix, TR/TE = 8325/86 ms, b = 1000 s/mm^2^, number of b0s = 2).
5. Post-contrast (gadolinium) Sagittal 3D T1: GE BRAVO sequence, duration 4 min each, FOV 256mm, Slice thickness 1mm, TE 2.7ms, TR 7.2ms, Flip angle 12°, Pixel spacing 1mm. Acquisition Matrix (Freq x Phase) is 256x256, which results in 1mm isotropic acquisition voxel size. The reconstruction matrix is 256x256.
6. MD-based normalisation algorithm comprising following steps was developed and implemented in Phyton:
7. For each pair of lesional/non-lesional streamlines two marginal points on each side of the streamline (points 1,2 and 12,13) were selected for lesional and non-lesional streamlines on MD, AD and RD plots and fitted with linear function (Suppl Fig. 2a. lesional fibers-blue arrow, non-lesional fibers-red arrow).
8. Difference between lesional and non-lesional slopes were calculated for each diffusion modality i.e. slope of ΔAD, ΔRD and ΔMD (Suppl Fig. 2b)
9. Slop of ΔMD function was subtracted from slops of ΔAD and ΔRD functions
10. Residual slopes were used to correct initial AD and RD values along entire lesional streamline.
