# Supplementary material for: Differentiating axonal loss and demyelination in chronic MS lesions: A novel approach using single streamline diffusivity analysis
Source: PLoS One. 2021 Jan 6;16(1):e0244766. doi: 10.1371/journal.pone.0244766 (PMC7787472; doi:10.1371/journal.pone.0244766)
Supplement: Attachment — Submitted filename: reply to reviewers 2.docx [file pone.0244766.s005.docx]

Please find response to reviewers’ comments below.

Reviewer #1: Authors have addressed properly all my comments and doubts.

Reviewer #2: The authors have made substantive changes to the manuscript so that their work has become much clearer. I have two issues, which I believe can easily be corrected, with the manuscript as it stands.
Q1. The authors have a whole bunch of metrics which have been shown in their studies, or in others, to correlate with various pathologies. Yet, the authors do not do the correlations themselves with the people in this study. The manuscript itself does an excellent job not overstating what was done. The abstract, on the other hand, does overstate what was done which can be dangerous as the abstract is often the only part of a manuscript that is read by researchers who are looking for some trick to describe their own data. Thus, I suggest changing the overreaching statement “Our result demonstrates close association between an increase of AD and the degree of axonal loss and support the notion that the increase of RD in chronic MS lesions is driven by the combination of two factors: axonal destruction and loss of myelin sheaths in survived axons. While the effect of demyelination dominates in cases of mild axonal loss, neurodegeneration is a primary cause of increased RD in more destructive lesions. These finding highlight the importance of selecting appropriate patient cohorts for clinical trials of pro-remyelinating and neuroprotective therapeutics.” to something more accurate. The summary the authors wrote at the end of the manuscript has a nice statement that could be used instead, “Evaluation of lesions in a sizable cohort of MS patients using the proposed method supports the use of ΔAD as a marker of axonal loss; and the notion that demyelination and axonal loss independently contribute to the increase of RD in chronic MS lesions.”

A1. Follow reviewer suggestion the last paragraph was removed from Abstract and replaced with following :” Evaluation of lesions in a sizable cohort of MS patients using the proposed method supports the use of ΔAD as a marker of axonal loss; and the notion that demyelination and axonal loss independently contribute to the increase of RD in chronic MS lesions”.

2. Once again the plots, while much more clear, are not in the standard format for plots. The authors seem to be confusing the title of the plot with what should be on the y-axis. This is true for almost every plot in the figures presented. To make things clear, I will explain what I mean using just the top graph of Figure 2b. There should be no title. The y-axis should be labelled AD (µm2/ms). The x-axis is labelled sufficiently with streamline.

All plots are modified as suggested.
